# The Role of Brown Adipose Tissue in Cardiovascular Disease Protection: Current Evidence and Future Directions

**DOI:** 10.15344/2456-8007/2019/136

**Published:** 2019-09-14

**Authors:** Renata O. Pereira, Samy I. McFarlane

**Affiliations:** 1 Department of Internal Medicine - Endocrinology and Metabolism, FOE Diabetes Research Center, University of Iowa, Iowa City, IA 52242, USA; 2 Department of Internal Medicine, State University of New York, Downstate Medical Center, Brooklyn, NYC 11203, USA

**Keywords:** Brown adipose tissue, Obesity, Cardiovascular disease, Batokines, Adipokines, Lipidomic cardioprotection

Obesity is a major public health problem that has reached epidemic proportions in the United States and around the globe. According to data from the Center for Disease Control (CDC), the prevalence of obesity was 39.8% and affected about 93.3 millions of US adults in 2015~2016, with nearly two thirds of Americans being either obese or overweight [1].

This rapid rise in obesity has also led to a concurrent rise in type 2 diabetes, cardiovascular disease (CVD), including heart disease and stroke, as well as cancer, resulting in increased morbidity and premature mortality among affected individuals [2]. The major CVD effects of obesity, particularly central obesity, are mediated through traditional as well as non-traditional CVD risk factors (table 1a and 1b) that include multiple components of the metabolic syndrome such as insulin-resistance, dyslipidemia, and hypertension [3]. The most effective therapies at reversing cardiovascular disease (CVD) risk factors associated with obesity have been dietary changes and exercise [4]. However, sustainable adherence to these lifestyle interventions has proven to be challenging and control of CVD risk factors in the diabetic population is largely suboptimal and only achieved concomitantly in about one third of the patients [5], therefore, novel therapeutic targets are desperately needed in order to combat the rising epidemic of obesity and its consequent increase in diabetes and CVD.

Among the novel potential therapeutic targets, brown adipose tissue (BAT) has generated substantial interest since the discovery of functional BAT in adult humans. Upon stimulation by cold exposure or other stimuli, BAT is activated to promote non-shivering thermogenesis, thereby contributing to increased energy expenditure. The major inducer of BAT growth and activation is the sympathetic nervous system, through the release of norepinephrine [6]. Classically, it was believed that functional BAT only existed in humans in the infant stage; however, functional BAT was recently identified in adult humans. Therefore, BAT activation has emerged as a potential strategy for elevating energy expenditure to counteract obesity and associated comorbidities, such as diabetes and CVD [7]. In addition, recent evidence in mice and humans suggest a role for BAT in regulating components of cardiovascular health, in a manner that may be independent of its thermogenic properties, via the secretion of endocrine factors [8].

High BAT activity correlates with high resting metabolic rates upon cold stimulation, and with low body mass index and low-fat mass [9–11]. However, the contribution of BAT activation to whole-body energy expenditure in humans has been under debate. Studies measuring BAT oxygen consumption or glucose uptake as a marker of BAT activity following acute cold exposure have used theoretical calculations to estimate BAT-induced energy expenditure. However, the data is extremely variable depending on the method used to estimate BAT activity and mass [12], with values ranging from 25 kcal/day to 211 kcal/day. Importantly, all these theoretical calculations assume a maximally activated BAT across a period of 24 hours, which is unlikely to ever occur [13,14]. Collectively, these data suggest thermogenesis due to activated BAT seems small and unlikely to lead to clinically meaningful reductions in body weight, even in the absence of compensatory increases in energy intake. Accordingly, no change in body weight has been observed in human subjects exposed to 19 °C for 10 h/d during 4 weeks [15], or after exposure to 17 °C for 2 h/d during 6 weeks [16]. Additionally, although BAT is more active upon low outdoor temperatures, obesity prevalence is not associated with outdoor temperature once adjusted to poverty, race, and education [17]. Hence, activation of BAT and its resulting increase in energy expenditure appears insufficient as a potential strategy for the treatment of obesity in humans. Nonetheless, the role of BAT activation as a means to prevent long-term weight gain and to assist in weight management after weight loss warrants further investigation. Moreover, activation of BAT may improve overall metabolic health and increase substrate utilization [18].

Hypothetically, the high metabolic activity of BAT could be beneficial for increasing the clearance and utilization of circulating glucose and lipids, hence potentially ameliorating systemic metabolic homeostasis. This hypothesis is supported by indirect evidence demonstrating that low outdoor temperature is associated with high BAT activity [19, 20], lower prevalence of glucose intolerance and lower incidence of type 2 diabetes, independent of age and obesity [21]. Presence of detectable BAT was also associated with lower circulating HbA1c and glucose concentrations after controlling for age, sex and body fat mass [22]. Thus, several studies analyzed the effect of cold-induced BAT activation on glucose homeostasis and lipid metabolism.

Acute cold exposure was reported to increase basal and insulin-stimulated whole-body glucose disposal in individuals with high BAT mass and activity [23]. Similarly, 10 days of daily cold exposure increased insulin-stimulated glucose disposal in individuals with type 2 diabetes [24]. Taken together, these studies suggest cold-induced BAT activation improves glycemic control. However, recent calculations, [13] suggest that the observed beneficial effects in glucose homeostasis in response to cold seem mostly due to a metabolic effect in skeletal muscle rather than in BAT [25]. Regarding lipid metabolism, acute cold exposure has been shown to increase BAT non-esterified fatty acids (NEFA) uptake and appearance into circulation [26]. A separate study reported a decrease in total circulating levels of cholesterol and LDL-cholesterol among individuals with hypercholesterolemia following chronic cold exposure for 90 days [27]. However, the effects of cold exposure on circulating lipid levels do not necessarily depend exclusively on BAT activity. Actually, BAT explains only 0.5% of plasma NEFA turnover following acute cold exposure [28].

In addition to studies using cold exposure as a means to increase BAT activity, several studies were performed using pharmacological approaches to activate BAT. Catecholamines are physiological humoral factors that activate BAT through β3-adrenoreceptors. Therefore, studies investigating the role of the acute and chronic effects of β3-adrenoreceptor agonists on glucose and lipid homeostasis have been performed. Acute administration of mirabegron or TAK-677, β3-adrenoreceptor agonists, showed no effect on glycemia [29,30]. Moreover, circulating NEFA concentration was not modified by acute administration of mirabegron, but increased after TAK-677 [30]. Chronic administration of TAK-677 for 29 days [30] or CL316, 243 for 8 weeks [31] showed no effect on glycemia, but CL316, 243 did increase insulin-mediated glucose disposal. These compounds had divergent effects on blood NEFA concentration, with TAK-677 showing no effect [30], whereas NEFA concentration was elevated after CL316, 243 administration [31]. Thus, treatment with β3-adrenoreceptor agonists appears ineffective to reduce blood glucose and lipid concentrations, despite the evidence showing enhancement in insulin-mediated glucose disposal [31]. Furthermore, cardiovascular safety is of concern given the increase in heart rate and blood pressure observed with this class of medication [32]. Therefore, studies investigating new pathways that can be targeted to induce BAT’s activity, independently of β3-adrenergic activation are of great significance, and may give rise to more efficacious and safer treatments to counteract obesity and its comorbidities.

Recent studies have uncovered an additional role for BAT as an endocrine organ [33]. Brown adipose tissue secretes several molecules, which are collectively termed batokines. Indeed, studies using transplantation of BAT in rodents have shown beneficial effects on metabolism and cardiovascular outcomes, which can be explained by the release of these endocrine factors into the circulation. For instance, BAT transplantation reversed the glycemic symptoms of two different models of type 1 diabetes without a change in insulin levels [33,34]. These batokines may alter metabolism by autocrine, paracrine, and endocrine mechanisms, thus modifying BAT itself or acting remotely on other organs. Some batokines have been shown to act as endocrine factors with potential beneficial roles in cardiovascular health, either by improving systemic metabolism or by directly affecting the heart (Table 2).

The first known endocrine factor secreted by BAT was the active thyroid hormone triiodothyronine (T_3_), which is elevated in the plasma in response to BAT activation. Thyroid hormones were shown to exert strong cardioprotective effects in both humans and animals [35], despite their deleterious chronotropic effect. However, the active cardioprotective role of BAT-mediated T_3_ production is uncertain, since T_3_ is produced by many other tissues [36]. Neuregulin 4 (Nrg4) is another factor that is strongly induced during brown adipogenesis and by cold-induced BAT activation. Nrg4 protects against diet-induced insulin resistance and hepatic steatosis via attenuation of lipogenic signaling [37]. Regarding cardiovascular health, Nrg4 was shown to be secreted by the liver in a myocardial ischemia (MI) model. In this study, Nrg4 had cardioprotective effects against MI injury when administered to mice [38]. However, whether Nrg4 is sufficiently released from BAT under conditions of cardiomyocyte injury to provide cardiovascular protection is unknown.

BAT-derived IL-6, a proinflammatory cytokine, has been shown to mediate glucose metabolism and energy balance. Studies using BAT transplantation promoted improvements in glucose homeostasis and insulin sensitivity which were eliminated when BAT from IL-6 KO mice was transplanted [39], suggesting that IL-6 mediated the beneficial effects of BAT transplantation. However, because of its complex signaling and ubiquitous synthesis by many tissues, the therapeutic relevance of IL-6 seems unlikely [40]. Furthermore, IL-6 was shown to have an acute cardioprotective effect role in heart failure, whereas long-term exposure contributes to maladaptive cardiac remodeling and contractility [41].

Another endocrine factor secreted by cold-activated BAT is fibroblast growth factor 21 (FGF21). FGF21 exerts beneficial effects on lipid and glucose metabolism in mice and humans, and was identified as a candidate endocrine factor released by transplanted BAT [39]. FGF21 is considered a promising new therapy to reduce obesity and associated comorbidities, both by activating BAT and by acting on white adipose tissue (WAT) and the liver [42]. Importantly, FGF21 was recently reported to have both antihypertrophic and cardioprotective actions in animal models of hypertrophy [43] and ischemia [44]. It is noteworthy, though, that secretion of FGF21 is not specific to BAT; other tissues, including liver [45] and skeletal muscle [46], can also release FGF21 into the circulation. Nevertheless, FGF21 remains a topic of interest for the treatment of hypertrophy and ischemic injury of the heart.

Recent studies have identified yet another endocrine factor that can be secreted from BAT in response to cold [47] and caloric excess [48], growth and differentiation factor 15 (GDF15). GDF15 is a circulating protein that has been implicated in regulation of energy homeostasis, body weight and cachexia. The potential to target GDF15 in the treatment of energy-intake disorders, including obesity and anorexia, is an area of intense investigation. GDNF family receptor a-like (GFRAL) was recently identified as the neuronal receptor responsible for mediating the anorectic actions of GDF15 [49]. However, the mechanisms by which GDF15 mediates its additional effects on metabolism are incompletely understood. Regarding the role of GDF15 in cardiovascular health, studies in rodents suggest GDF15 may have a protective role in agonist-induced hypertrophy, ischemia/reperfusion injury [50,51], and in atherosclerosis [52]. In addition, a recent study demonstrated that GDF15 stimulates hepatic triglyceride export via beta-adrenergic signaling in mice, which allows for maintenance of triglyceride levels, thereby conferring cardioprotection during acute inflammation [53]. In humans, GDF15 has been shown to be associated with cardiovascular dysfunction and is over expressed in the myocardium of patients with MI [54]. However, the role of GDF15 on CVD protection in humans is unclear.

Several lipids have been identified that are released from tissues and act locally or systemically to promote insulin sensitivity and glucose tolerance; as a class, these lipids are referred to as lipokines. Upon cold-induced activation of BAT, free fatty acids (FFA) are released from stored triglycerides (TG) by lipolysis. Therefore, it was hypothesized that there might be thermogenic lipokines that activate BAT in response to cold. Indeed, a study using global lipidomics found that the lipid 12,13-dihydroxy-9Z-octadecenoic acid (12,13-diHOME) was increased in the circulation of humans and mice exposed to cold. Injection of 12,13-diHOME acutely activated BAT fuel uptake and enhanced cold tolerance. Mechanistically, 12,13-diHOME increased fatty acid (FA) uptake into brown adipocytes by promoting the translocation of FA transporters to the cell membrane [55]. On a separate study, secretion of 12,13-diHOME from BAT was found to be increased in response to a single bout of exercise in humans and rodents, which was associated with increased fatty acid oxidation and uptake in skeletal muscle. These data suggest that 12,13-diHOME, or a functional analog, could be developed as a treatment for metabolic disorders [56]. A study in isolated murine hearts suggest 12,13-diHOME may decrease post-ischemic cardiac recovery [57], however, the role of 12,13-diHOME in cardiovascular health in humans is unknown.

Finally, a study suggested that BAT is activated and exerts systemic cardioprotective effects in models of catecholamine-induced injury in mice, leading to decreased myocardial injury, fibrosis, and pathological left ventricle remodeling. The authors suggest this cardioprotective effect could be due to systemic actions of BAT-derived secreted factors, although no specific batokines were identified in this study [58].

## Conclusion

We presented the current knowledge suggesting a general beneficial effect of BAT activation toward the reduction in CVD risk. We also discussed BAT-secreted factors with potential direct and/or indirect cardioprotective effects via modulation of systemic metabolism. Studies of different models of cardiac stress would be of value in determining if BAT activity is also induced and whether it is associated with improved cardiac phenotypes. Determining the specific batokines mediating the cardioprotective effects would also offer valuable insights regarding the potential role of BAT on cardiovascular protection. These secreted factors may exert metabolic benefits on adipose tissues, the heart, and /or other peripheral tissues in addition to coordinating the metabolic adaptations during cardiovascular insults, thus potentially resulting in cardio protection.

Finally, understanding the mechanisms regulating induction of batokines could result in identification of useful therapeutic targets based on brown fat activation. Future research is warranted to address some of these questions and to determine if BAT and its batokines will yield novel therapeutic options for the treatment of the growing epidemic of obesity and associated metabolic disorders, and CVD.

## Figures and Tables

**Table 1a:** Traditional risk factors for cardiovascular disease.

1.	Advanced age
2.	Male gender
3.	Post-menopausal state
4.	Cigarette smoking
5.	Hypertension
6.	Insulin resistance / diabetes mellitus
7.	Intra-abdominal obesity
8.	Dyslipdemia including: low level of high-density lipoprotein, high triglyceride levels and small dense low-density lipoprotein

**Table 1b:** Non-traditional risk factors for cardiovascular disease.

1.	Microalbuminuria
2.	Endothelial dysfunction
3.	Increased C-reactive protein and other inflammatory markers
4.	Increased Apo-lipoprotein B levels
5.	Increased LPa
6.	Increased fibrinogen levels
7.	Increased plasma activator inhibitor-1 level (PAI-1)
8.	Absence of nocturnal dipping in blood pressure and pulse (non-dippers)
9.	Salt sensitivity
10.	Left ventricular hypertrophy
11.	Hyperhomocystienemia

**Table 2:** BAT-secreted factors with potential direct and/or indirect cardioprotective effects via modulation of systemic metabolism.

BAT-Secreted Factor	Systemic and Cardiovascular Actions	Ref. #
T_3_	Improves systemic metabolism Cardioprotective effect Chronotopic effect	[35,36]
Nrg4	Decreases insulin resistance Cardioprotective effect	[37,38]
Il-6	Improves systemic metabolism Acute cardioprotective effect Maladaptive remodeling (chronic effect)	[39–41]
FGF21	Improves systemic metabolism Anti-hypertrophic effect Cardioprotective effect in MI	[39,42–44]
GDF15	Improves systemic metabolism Cardioprotective in hypertrophy, I/R injury, atherosclerosis and acute inflammation	[49–53]
12,13-diHOME	Increases fuel uptake in BAT and muscle Decreases post-ischemic recovery	[55–57]

T3 = triiodothyronine, Nrg4 = Neuregulin 4, Il-6 = interleukin-6, FGF21= fibroblast growth factor 21, GFD15 = Growth differentiation factor 15, 12,13-diHOME = 12,13-dihydroxy-9Z-octadecenoic acid.
